# The Impact of IncontiLase Er:YAG Laser Treatment on the Quality of Life of Women With Stress Urinary Incontinence

**DOI:** 10.1155/aiu/1255593

**Published:** 2025-12-03

**Authors:** Vidya Pancholia, Tamanna Pancholia, Iva Talaber, Zdenko Vižintin

**Affiliations:** ^1^ Vcare Laser Centre for Cosmetic Gynecology, Indore, Madhya Pradesh, India; ^2^ Laser and Health Academy, Ljubljana, Slovenia

**Keywords:** Er:YAG, IncontiLase, patient-reported outcome, quality of life, stress urinary incontinence

## Abstract

**Objective:**

Assessment of health‐related quality of life (QoL) is particularly crucial in urinary incontinence because this condition significantly affects patients’ social, psychological, physical, emotional, and economic wellbeing. The objective of this service evaluation was to assess various QoL outcomes in patients with stress urinary incontinence (SUI) before and after treatment with a nonablative Er:YAG laser.

**Methods:**

Upon receiving SUI laser treatment (IncontiLase) at our clinic, 49 patients subsequently provided follow‐up assessments of health‐related QoL outcome measures at two time points (2‐month FU (FU1) and 9‐month FU (FU2)).

**Results:**

Prior to treatment, patients were strongly bothered by their SUI symptoms. Two months after treatment, all the patients reported significant improvements of SUI symptoms and SUI‐related QoL, which continued to improve with time. According to the QoL scores, patients did not consider their SUI symptoms bothersome enough to seek further medical care after laser treatment, even after a period of 9 months.

**Conclusions:**

Nonablative Er:YAG laser treatment was associated with substantial, clinically meaningful improvements in patient‐reported SUI‐related QoL in a real‐world clinical setting. This service evaluation supports the use of Er:YAG laser treatment in clinical practice, although the study design limits the strength of conclusions regarding its effectiveness.

## 1. Introduction

Stress urinary incontinence (SUI) remains a prevalent yet underdiagnosed and underreported condition, with an estimated 200 million individuals, mostly women, affected worldwide. Age is the single largest risk factor for urinary incontinence. Despite its significant impact on quality of life (QoL), 50%–70% of women with urinary incontinence do not pursue treatment [1], due to social stigma, and the belief that it is a “normal” part of aging [2]. In addition to the physical impact, urinary incontinence is associated with emotional and social consequences such as anxiety, depression, work impairment, and sexual dysfunction, all of which reduce women’s QoL [3].

SUI is most often associated with weakened tissues of the pelvic floor and consequent hypermobility of the bladder neck and urethra. Leakage of urine occurs with increases in intra‐abdominal pressure such as with coughing, laughing, sneezing, and exercise [4]. Aside from advancing age, other risk factors for SUI include coexisting morbidity, cognitive dysfunction, functional impairment, gait abnormality, diuretic therapy, and obesity [5]. With the growing elderly population, the prevalence of urinary incontinence, particularly among women, is expected to rise significantly.

Therapeutic management options include pelvic floor physiotherapy, anti‐incontinence devices, surgery, and laser therapy. Treatment with nonablative Er:YAG laser aims to improve pelvic floor support by stimulating collagen production in the vaginal wall, thereby enhancing connective tissue strength to improve the urethral support [6]. The rationale behind this form of treatment is based on collagen reconstruction, which consecutively assumes its role in providing vaginal support to pelvic floor structures [7]. More specifically, nonablative Er:YAG laser therapy results in controlled heating of the underlying mucosal layers, without burning the mucosa [8] or ablating tissue. The Er:YAG laser ablates soft tissue at fluences above ∼2 J/cm^2^ [9]. The IncontiLase technique uses nonablative, subthreshold fluences delivered in a pulse‐stacking sequence. When pulses are spaced to allow superficial cooling (Ts ≈ 10 ms) but delivered faster than the deeper tissue’s thermal relaxation time (Tb ≈ 1–2 s), heat accumulates in deeper layers [10]. The subsequent thermal injury of the tissue [8, 11–15] results in a reactive inflammatory response and an increase of the biosynthetic capacity of fibroblasts and other cells, inducing the reconstruction of an optimal physiological environment, the enhancement of cell activity, hydration, and the synthesis of collagen and elastin ([16, 17]; [10, 18–21]). A recent meta‐analysis of RCT studies with a total of 390 SUI patients concluded that Er:YAG laser therapy is a safe and effective treatment option for SUI [22].

Much of the available studies on SUI treatment focus on clinical outcomes, which do not assess an individual’s satisfaction and the feeling of wellbeing following therapeutic intervention and hence may have limited relevance to women with SUI [3]. On the other hand, a patient‐reported outcome is a measurement of any aspect of a patient’s health that comes directly from the patient, without interpretation of the patient’s response by a physician or anyone else [23]. In the context of female urinary problems, various QoL tools have been designed and investigated. QoL refers to the degree to which a person enjoys the possibilities of his or her life. It is a reflection of an individual’s sense of wellbeing and satisfaction with life [24]. SUI can be a frustrating and embarrassing issue for women, often disrupting daily activities like exercise, laughing, or even sneezing. The constant worry about leaks can lead to reduced confidence, social withdrawal, and interference with everyday comfort and routines. The effects of SUI on overall health status and health‐related QoL [25] are efficiently assessed with the King’s Health Questionnaire (KHQ) and the Urogenital Distress Inventory (UDI‐6) questionnaires due to their strong psychometric properties and ease of administration [24].

The aim of this service evaluation was to assess patient‐reported QoL outcomes following nonablative Er:YAG laser treatment IncontiLase in women with SUI.

## 2. Methods

This was a service evaluation which included consecutive SUI patients who initiated laser treatment at our clinic in India between January 1, 2019, and December 31, 2022. We aimed to include enough patients to be able to detect significant differences in QoL outcomes postintervention compared with baseline with a significance level of 0.05 and a statistical power of 0.8, which was estimated in a similarly designed study to be *n* = 31 [26]. Prior to laser treatment, patients were subjected to a standard gynecological and urine culture examination.

### 2.1. Laser Treatment

All patients were treated with nonablative Er:YAG laser treatment for SUI (IncontiLase, Fotona). The IncontiLase protocol lasts 20 min and consists of three steps.

1. Intravaginal laser pulses with a directed angular, patterned laser beam (PS03‐GAc, 7 mm, 6 J/cm^2^, 2.0 Hz, seven pulses, six positions, one pass per position),
2. Intravaginal laser pulses with a circular full laser beam (R11‐GCc, 7 mm, 3 J/cm^2^, 2.0 Hz, seven pulses, two passes),
3. Laser pulses of vestibule and introitus with a straight, patterned laser (PS03, 7 mm, 10 J/cm^2^, 1.6 Hz, two to three pulses, two to three passes, 10% overlapping).

The treatment consisted of two or three IncontiLase sessions with 1‐month interval.

### 2.2. Patient‐Reported Assessment of QoL

Patient‐reported outcome assessment was documented with two validated questionnaires: UDI‐6 and KHQ. Questionnaires were administered before the laser treatment (at baseline) and 2 months (FU 1) and 9 months (FU 2) after completed treatment.

While both the KHQ and UDI‐6 questionnaires are used to assess the impact of urinary incontinence, the UDI‐6 focuses primarily on symptom distress—specifically, the degree of discomfort or bother caused by symptoms—whereas the KHQ captures the broader impact on various aspects of daily life. As a more comprehensive tool, the KHQ requires significantly more time to complete, which is why it is not routinely administered in clinical studies.

The UDI consists of 6 items: 1: Frequent urination, 2: Leakage related to feeling of urgency, 3: Leakage related to activity, 4: Coughing or sneezing small amounts of leakage (drops), 5: Difficulty emptying the bladder, and 6: Pain or discomfort in the lower abdominal or genital area. Higher scores in UDI‐6 indicate higher disability. Total score is from 0 to 100 [25].

The KHQ is a tool recommended by the European Clinical Practice Guidelines [27] and consists of 30 questions [24], divided into 9 domains (1: General health, 2: Incontinence impact, 3: Limitation of Daily Activities, 4: Physical limitations, 5: Social limitations, 6: Personal relationships, 7: Emotions, 8: Sleep/Energy, and 9: Severity Measures) that address the impact of UI on QoL. The score ranges from 0 to 100 per domain, with a higher score indicating lower QoL in that domain.

### 2.3. Ethical Standard

Ethical approval was not sought because service evaluation does not require specific approval from a research ethics committee or IRB [28]. Nevertheless, ethical principles were still adhered to for the protection of patients.

#### 2.3.1. Informed Consent

All participants provided written informed consent prior to treatment and participation in the study. They were fully informed about the study’s purpose, the type and scope of data to be collected and the intended use of their anonymized QoL data.

#### 2.3.2. Anonymization and Confidentiality

All QoL data were fully anonymized prior to analysis and publication. The study design included robust safeguards for confidentiality of personal information and data security throughout collection, storage, and reporting processes. Because anonymized data cannot be traced back to individual participants, the study does not involve identifiable private information.

#### 2.3.3. Minimal Risk to Participants

The study posed no risk—physical, psychological, legal, or social—to participants: it involved noninvasive data collection (QoL surveys), participants were paying clients who voluntarily sought treatment and no deviation from standard clinical care occurred for the service evaluation purposes.

### 2.4. Statistics

Statistical analyses were performed in GraphPad Prism. The distribution of the data was assessed using the Shapiro–Wilk test. As normality was not met, nonparametric tests were performed. A nonparametric Friedman test of differences among repeated measures (time points) was conducted. To analyze which pairs of repeated measures (time points) are significantly different from each other the post hoc Dunn’s multiple comparisons test was used. Statistical significance level in the figures was denoted as i) ^∗^: *p* < 0.05, ii) ^∗∗^: *p* < 0.01, iii) ^∗∗∗^: *p* < 0.001, and iv) ^∗∗∗∗^: *p* < 0.0001.

## 3. Results

**Figure 1:**
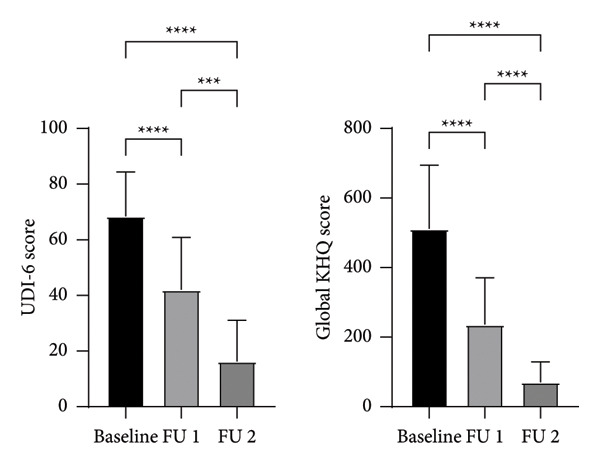
UDI‐6 (left) and global KHQ (right) scores reported by the patients (*n* = 49) at different time points (baseline, 2‐month FU (FU 1), and 9‐month FU (FU 2)).

**Figure 2:**
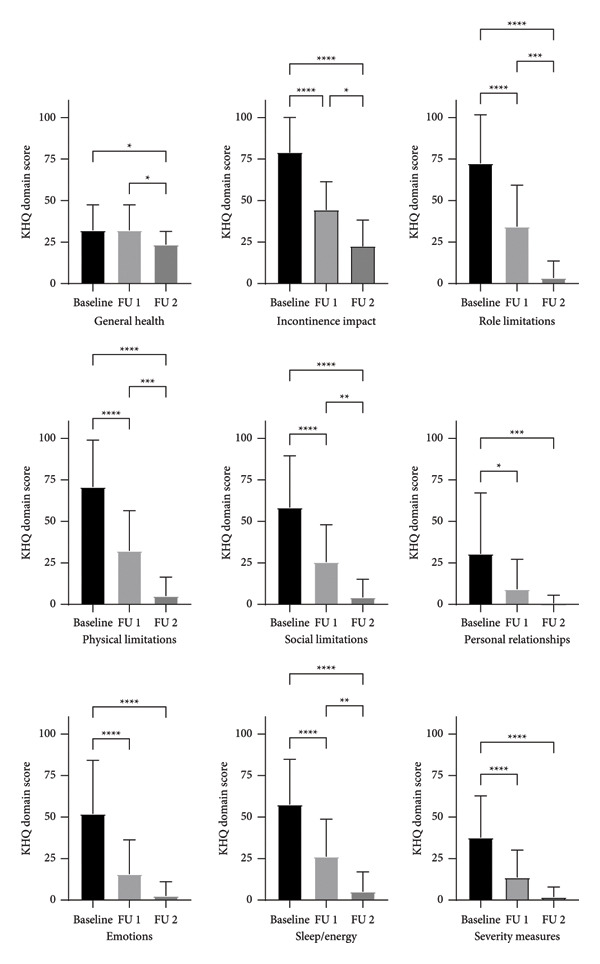
Specific KHQ domain scores reported by the patients (*n* = 49) at different time points (baseline, 2‐month FU (FU 1), and 9‐month FU (FU 2)).

A total of 49 female patients of ages 28—84 years (mean: 49.9 ± 12.8) with SUI were included in the study. The QoL outcomes of 49 patients are summarized in Tables 1 and 2. No significant correlation between age and KHQ or UDI‐6 scores was found (data not shown), so the QoL data were not stratified according to patient age. Both the UDI‐6 and global KHQ scores decreased significantly (*p* <  0.0001) after treatment, both at FU 1 and FU 2 (Figure 1). Mean change from baseline in the UDI‐6 score at FU 1 was 26.5 (Table 1). This value is higher than the reported minimal clinically important difference (MCID) for UDI‐6, defined as a decrease of ≥ 16.7 points [29]. All KHQ domains demonstrated significant improvements over the duration of assessment (Table 2), with significant difference between pairs of time points shown in Figure 2. Mean change from baseline in the KHQ score at 2‐month FU in every KHQ domain was higher than MCID for KHQ, defined as a decrease of 10 points [30].

**Table 1:** Results of the Urogenital Distress Inventory (UDI‐6) questionnaire.

UDI‐6	Baseline	2‐month FU (FU 1)	9‐month FU (FU 2)	Group comparison	
				Friedman statistic	*p* value
*Total score*					
Mean ± SD	68.5 ± 15.9	42.0 ± 18.9	16.3 ± 14.8	92.54	*p* < 0.0001
Median (min; max)	70.8 (54.2; 79.2)	41.7 (25.0; 54.2)	16.7 (0.0; 50.0)		
Mean change from baseline	/	26.5	52.2		

**Table 2:** Results of the King’s Health Questionnaire (KHQ).

KHQ domain	Baseline	2‐month FU (FU 1)	9‐month FU (FU 2)	Group comparison	
				Friedman statistic	*p* value
*General health*					
Mean ± SD	54.6 ± 23.8	32.1 ± 15.3	23.5 ± 7.9	34.00	*p* < 0.0001
Median (min; max)	50.0 (0.0; 100.0)	25.0 (0.0; 75.0)	25.0 (0.0; 50.0)		
Mean change from baseline	/	22.5	31.1		

*Impact of incontinence*					
Mean ± SD	78.9 ± 21.2	44.2 ± 17.2	22.5 ± 15.8	84.96	*p* < 0.0001
Median (min; max)	66.7 (33.3; 100.0)	33.3 (0.0; 66.7)	33.3 (0; 33.3)		
Mean change from baseline	/	34.7	56.4		

*Limitation of daily activities*					
Mean ± SD	72.5 ± 29.2	34.6 ± 24.9	3.4 ± 10.2	88.19	*p* < 0.0001
Median (min; max)	66.7 (16.7; 100)	33.3 (0.0; 66.7)	0.0 (0.0; 33.3)		
Mean change from baseline	/	37.9	69.1		

*Physical limitations*					
Mean ± SD	70.9 ± 28.2	32.3 ± 24.2	5.1 ± 11.4	86.21	*p* < 0.0001
Median (min; max)	66.7 (0.0; 100.0)	33.3 (0.0; 66.7)	0.0 (0.0; 33.3)		
Mean change from baseline	/	38.6	65.8		

*Social limitations*					
Mean ± SD	58.4 ± 31.1	25.5 ± 22.5	4.3 ± 10.8	76.49	*p* < 0.0001
Median (min; max)	55.6 (11.1; 100.0)	22.2 (0.0; 66.7)	0.0 (0.0; 33.3)		
Mean change from baseline	/	32.9	54.1		

*Personal relationships*					
Mean ± SD	42.2 ± 36.4	14.4 ± 17.7	1.5 ± 4.8	41.88	*p* < 0.0001
Median (min; max)	33.3 (0.0; 100.0)	0.0 (0.0; 66.7)	0.0 (0.0; 33.3)		
Mean change from baseline	/	27.8	40.7		

*Emotions*					
Mean ± SD	51.9 ± 32.2	15.7 ± 20.7	2.5 ± 8.6	77.06	*p* < 0.0001
Median (min; max)	66.7 (0.0; 100.0)	0.0 (0.0; 66.7)	0.0 (0.0; 33.3)		
Mean change from baseline	/	36.2	49.4		

*Sleep/Energy*					
Mean ± SD	57.5 ± 27.2	26.2 ± 22.6	5.1 ± 11.9	85.48	*p* < 0.0001
Median (min; max)	66.7 (0.0; 100.0)	16.7 (0.0; 66.7)	0.0 (0.0; 66.6)		
Mean change from baseline	/	31.3	52.4		

*Measures of severity*					
Mean ± SD	37.6 ± 25.1	13.6 ± 16.5	1.9 ± 6.0	80.82	*p* < 0.0001
Median (min; max)	33.3 (0.0; 100.0)	0.0 (0.0; 66.7)	0.0 (0.0; 33.3)		
Mean change from baseline	/	24	35.7		

*Global KHQ score*					
Mean ± SD	511.3 ± 182.8	232.8 ± 134.5	68.9 ± 57.5	97.51	*p* < 0.0001
Median (min; max)	536.1 (141.7; 825.0)	230.6 (11.1; 500.0)	58.3 (0.0; 258.3)		
Mean change from baseline	/	278.5	442.4		

## 4. Discussion

This service evaluation corroborates the negative correlation of UI and patient QoL demonstrated in previous studies, attributed to the social, psychological, physical, and emotional consequences of SUI, as well as its economic burden [31].

Patients included in this service evaluation were on average strongly bothered by their SUI symptoms, but after the IncontiLase treatment, their health‐related QoL significantly improved, and the observed improvement was clinically meaningful based on the MCID for UDI‐6 score [29]. This agrees with several studies reporting on the beneficial effect of the IncontiLase treatment for SUI [32–34]. While there is a general agreement among systematic reviews and meta‐analyses that vaginal laser therapy can improve SUI symptoms in the short term, the overall certainty of evidence is low, due to the heterogeneity of data, owing to inclusion of various types of lasers and various study designs [35–38]. Nevertheless, the most recent systematic review [22], which included only randomized controlled trials using nonablative Er:YAG laser treatment for SUI, concluded that it is a safe and effective therapy, although noting the evidence is limited by the small number of available studies. A similar decrease in UDI‐6 scores (approx. 40% decrease from baseline) in the short‐term period of up to 3 months after treatment has previously been shown for the Er:YAG laser treatment [39], as well as the fractional bipolar radiofrequency therapy [40], midurethral sling [41], electromagnetic stimulation therapy [42, 43], and Kegel exercises [42, 44]. While the UDI‐6 scores remained above the threshold indicative of symptomatic urinary incontinence [45] at the 2‐month follow‐up (FU1), they decreased below the threshold value distinguishing care seekers from noncare seekers by the 9‐month follow‐up [45]. This indicates that even after 9 months following the IncontiLase treatment, patients did not perceive their SUI symptoms bothersome to the extent that would urge them to seek additional medical care. The additional improvement detected between the first and second follow‐ups may be attributed to the process of neocollagenesis, caused by the IncontiLase treatment and lasting up to 6 months after therapy [10, 46, 47].

In addition to reduced SUI symptoms, all patients observed a decrease in the extent to which their incontinence problems affected various dimensions of the quality of their life, as assessed by different domains of the KHQ questionnaire [24]. These include a General health domain which rates the overall wellbeing, influenced by current and past health, an Incontinence impact domain, which rates the degree of bother caused by incontinence in personal life, a Limitation of daily activities domain, which rates the impact on routine activities such as workplace responsibilities and household chores, a Physical limitations domain, which rates the impact on physical activities such as walking, running, and bending, a Social limitations domain, which rates the effect on relationships and social participation, a Personal relationships domain, which focuses on the relationship with a sexual partner, sex life, and marital harmony, an Emotions domain, which rates the emotional impact of issues like depression, anxiety, and loss of self‐esteem, a Sleep/Energy domain, which rates the degree of sleep deprivation due to bladder problems, and a Severity Measures domain, which rates the impact on daily functioning, such as wearing pads or worrying about urinary odor [24]. All patients reported a clinically meaningful improvement in each domain. The observed change in score from baseline for a specific KHQ domain is comparable to the improvement observed in patients 3 months after receiving a midurethral sling [48] and in patients 6 months after receiving IncontiLase in a recent RCT study [34]. Another RCT study demonstrated significantly greater decrease in KHQ scores in the group receiving IncontiLase treatment relative to sham [32]. Furthermore, the improvement in KHQ score observed in this study is higher [34, 48] or similar to [49] the improvement observed in patients performing pelvic muscle floor training for 3 months.

Notably, our evaluation of the health‐related QoL was based on the UDI‐6 and KHQ alone, which limits direct comparison of outcomes more commonly reported in clinical trials focusing on SUI symptom severity, i.e., the frequently used ICIQ‐SF. Nevertheless, the tools used in the present study enable assessment of how bothered patients feel by their symptoms, making it particularly suitable for capturing subjective improvements in a service evaluation setting. Furthermore, we acknowledge that detailed inclusion criteria, standardized diagnostic procedures, and stratification of SUI severity were not specified in this work. This reflects the design of the project as a service evaluation rather than a formal research study. The objective was to assess outcomes in routine clinical practice, where patient selection and diagnosis were based on clinician judgment and standard care pathways rather than predefined research criteria. While this limits direct comparability with randomized controlled trials, it provides meaningful real‐world data on the use and effectiveness of the intervention in everyday clinical settings.

Based on the results of this service evaluation, we can conclude that SUI patients experience a significant improvement in health‐related QoL after IncontiLase treatment. The strength of this service evaluation is a fully powered study sample and the comprehensive assessment of QoL at three time points. However, as this was a service evaluation without a control or sham comparator group, the potential contribution of placebo effects cannot be excluded. This is an important limitation, particularly given the known placebo response associated with SUI interventions. Furthermore, as all participants were self‐selected paying clients in a private clinic setting, selection bias cannot be excluded. These individuals may differ from the general population of women with SUI in terms of motivation, expectations, and health‐seeking behavior, which may have influenced the reported outcomes.

## Conflicts of Interest

Iva Talaber and Zdenko Vižintin are employed at Fotona d.o.o., the manufacturer of a medical device.

## Author Contributions

All authors contributed equally to this work.

## Funding

No funding was received for this manuscript.

## Data Availability

The data that support the findings of this study are available from the corresponding author upon reasonable request.
